# Supplementary material for: Effect of Atomized Black Maca (Lepidium meyenii) Supplementation in the Cryopreservation of Alpaca (Vicugna pacos) Epididymal Spermatozoa
Source: Animals (Basel). 2023 Jun 21;13(13):2054. doi: 10.3390/ani13132054 (PMC10339992; doi:10.3390/ani13132054)
Supplement: Supplementary file 1 [file animals-13-02054-s001.zip › animals-2424389-supplementary.pdf]

Supplemental Table S~~1.~~ Sperm parameters before (Fresh) and after freezing/thawing (*).

Groups	Motility %	Viability %	Membrane integrity (HOST%)	DNA fragmentation Index (%)	Sperm ROS+ (%)	Mitochondrial activity index
**Fresh**	76.2 ± 7.72[a]	61.25 ± 8.29[a]	69.16 ± 8.21[a]	1.64 ± 0.83[a]	29.2 ± 10.7[a]	0.89±0.26[a]
***Yolk Medium**	23.8 ± 5.28[b]	43.8 ± 4.83[b]	51.25 ± 7.72[b]	4.47 ± 3.10[a]	40 ± 12.6[b]	0,91 ± 0,28[a]
***Maca 10 mg/ml**	32.5±5 [c]	44.6 ± 3.96[b]	47.1 ± 6.20[b]	4.55 ± 1.91[a]	25.8 ±7.93[a]	0,896 ± 0,26[a]
***Maca 20 mg/ml**	40.4 ± 7.22[d]	48.8 ± 5.69[c]	58.33 ± 7.78[c]	3.95 ± 1.88[a]	19.2 ± 7.33[c]	0,882 ± 0,17[a]
***Maca 30 mg/ml**	22.1 ±5.42[b]	38.3 ±6.85[d]	52.1 ± 3.96[b]	4.16 ± 1.77[a]	30 ± 14.8[a]	0,765± 0,19[a]
***Resveratrol**	29.2 ± 6.34[c]	50.0 ±5.64[c]	57.5 ± 3.37[c]	4.90 ± 2.65[a]	22.5 ± 7.23[ac]	0.872 ± 0.09

Fresh: raw sperm; Yolk Medium: sperm cryopreserved in yolk medium (YM) ; Maca 10 mg/ml: YM supplemented with Maca 10 mg/ml; Maca 20 mg/ml: YM supplemented with Maca 20 mg/ml; Maca 30 mg/ml: YM supplemented with Maca 30 mg/ml; and Resveratrol: YM supplemented with Resveratrol 5mg/ml. Different superscripts within columns indicate significant differences ($P<0.05$).
